# A Retrospective Pragmatic Two‐Center Clinical Study to Evaluate the Clinical Outcome of Triple‐Frequency Ultrasound in the Treatment of Mild‐to‐Severe Acne Vulgaris

**DOI:** 10.1111/jocd.16672

**Published:** 2024-11-17

**Authors:** Irina G. Chervinskaya, Nataliia V. Gaidash, Ilja L. Kruglikov

**Affiliations:** ^1^ Clinic DK Forma St Petersburg Russian Federation; ^2^ Clinic TriActive Moscow Russian Federation; ^3^ Wellcomet GmbH Bruchsal Germany

**Keywords:** acne, retrospective pragmatic two‐center study, treatment, triple‐frequency ultrasound

## Abstract

**Objectives:**

Earlier, quickly alternating dual‐frequency ultrasound waves (LDM technology) were successfully applied for the treatment of different inflammatory skin conditions such as rosacea and acne. In this retrospective pragmatic two‐center clinical study, we applied the triple‐frequency LDM (TF‐LDM) technology with frequencies of 1/3/10 and 3/10/19 MHz for the treatment of mild‐to‐severe acne skin to assess the effectivity and sustainability of the treatment outcomes.

**Methods:**

Twenty‐two patients with mild‐to‐severe acne were included in this study: 11 patients were treated with TF‐LDM (1/3/10 MHz), and other 11 patients—with TF‐LDM (3/10/19 MHz). Assessment of the acne severity was done using the bilateral facial photographs. The photos were evaluated at baseline (T1), on the day of the last treatment (T2), and during the follow‐up controls (T3). Assessment of the acne severity was provided in accordance with a modified Global Evaluation Acne (mGEA) scale by nine independent dermatologists who were blinded to treatment assignments.

**Results:**

The average improvement of the mGEA scoring between T1 and T2 across all patients was 73.69% ± 13.90% (*p* < 0.01), whereas the skin improvement between T1 and T3 was 90.14% ± 8.35% (*p* < 0.01). The state of the skin was also statistically significantly improved between T2 and T3 (53.26% ± 29.24%, *p* < 0.02). There was no difference in treatment outcomes between the patients treated with TF‐LDM (1/3/10 MHz) and TF‐LDM (3/10/19 MHz).

**Conclusions:**

TF‐LDM is an effective method for the treatment of the mild‐to‐severe acne skin that provides a significant skin improvement and long‐lasting treatment results. The method demonstrates no significant side effects, is pain‐free, well tolerated, and highly accepted by patients.

## 1 Introduction

Acne vulgaris is the most common chronic inflammatory skin condition affecting almost all individuals at some point during adolescence or adult life and related to the malfunctioning of the pilosebaceous units (PSUs). Pathophysiology of acne vulgaris was connected with different etiopathogenic factors, for example, hyperseborrhea, follicular hyperkeratinization, and pathogenic behavior of the Gram‐positive bacterium *Cutibacterium acnes* [1]. Traditionally, hyperseborrhea was linked with increased sebum excretion from sebaceous gland induced by androgens; follicular hyperkeratinization was believed to be mainly caused by abnormal proliferation, terminal differentiation, and desquamation of keratinocytes; and the pathogenic behavior of *C. acnes* was related to uncontrolled proliferation of these bacteria in PSUs. On the other hand, inflammatory events are present in the very early stages of acne development and can be found both in involved and uninvolved acne skin, which allowed to consider acne vulgaris as a primary inflammatory skin disorder [2].

This pathophysiology of acne was substantially revised in the recent past after the discovery of the role of dermal adipocytes and caveolin‐1 (CAV1) in the development of inflammatory and hyperproliferative conditions in the vicinity of PSUs. Discovery of dermal adipocytes having the ability to produce the antimicrobial peptide cathelicidin during their reactive adipogenesis (quick and significant enhancement of adipogenesis in response to invading pathogens or their products such as lipopolysaccharides) turned the attention to a possible role of these cells (spatially located near the dermal–hypodermal junction and concentrated in the cone‐like structures around the PSUs) in inflammatory skin conditions [3, 4, 5]. Dermal adipocytes have a high plasticity and demonstrate reversible cyclic phenotypical transformations during the hair follicle (HF) cycle: They de‐differentiate in fibroblast‐like preadipocytes during the catagen phase and re‐differentiate in mature adipocytes during the anagen phase of the HF cycle [6, 7]. As we argued before, such phenotypic conversions of dermal adipocytes can significantly influence the development of acne through a local loading of the reticular dermis with proinflammatory lipids released during their de‐differentiation [4, 7]. This allowed to explain a long‐known predominant initiation of acne lesions in the catagen/telogen phases of the HF cycle [4]. Since under some pathological conditions the adipocyte‐derived preadipocytes can trans‐differentiate into myofibroblasts [7], we have earlier proposed that acne scarring should be considered as a collateral process tightly connected with these conversions [4].

Intensive research during the last years also clearly demonstrated the involvement of CAV1 in various inflammatory and hyperproliferative cutaneous conditions. This protein plays an important role in the stabilization of the plasma membrane invaginations named caveolae and is causally involved in multiple processes of cellular signaling. It was revealed that CAV1 is sufficiently reduced in such cutaneous conditions as psoriasis [8, 9], atopic dermatitis [10], and hypertrophic scarring/keloids [11], whereas its expression is strongly increased in the aged skin [12] and chronic wounds [13]. We have argued that CAV1 must be also strongly involved in the pathophysiology of acne and serve as a target in the treatment of this cutaneous condition [4]. Recently, it was reported that application of the state‐of‐the‐art treatment with 30% supramolecular salicylic acid in moderate‐to‐severe acne vulgaris indeed provided significantly increased expression of CAV1 in perilesional skin areas, and this expression negatively correlated with the release of inflammatory markers in the affected tissue [14].

Since CAV1 is not just a pathophysiological factor but also a target in various inflammatory skin conditions [4, 15], any methods that effectively modulate its expression in lesional/perilesional areas should be effective in the treatment of acne. It is well known that CAV1 expression can be enhanced by shear stress (e.g., through the application of ultrasound waves) and that this enhancement is frequency‐ and strain‐dependent [8]. For example, application of mechanical forces at frequencies of 1 MHz reduces the strain needed for the fluidization of cytoskeleton to about 10^−5^ of its value for the frequency of 1 Hz [16]. Moreover, it was demonstrated that application of higher ultrasound pressures and higher ultrasound frequencies induces higher levels of strain in the cells causing stronger fluidization of their cytoskeleton structure that is linked to caveolae [17]. This effect can be further enhanced by the application of ultrasound waves with very high frequencies over 10 MHz or by a quasi‐simultaneous application of different ultrasound waves known as the local dynamic micro‐massage (LDM) technology [18].

Earlier, application of ultrasound with a frequency of 10 MHz demonstrated a significant skin improvement in patients with acne and rosacea [19] as well as in other inflammatory cutaneous conditions [18]. In this retrospective pragmatic two‐center clinical study, we have applied the triple‐frequency LDM (TF‐LDM) waves with frequencies of 1/3/10 and 3/10/19 MHz for the treatment of mild‐to‐severe acne vulgaris to assess the effectivity and sustainability of the treatment outcomes.

## 2 Methods

### 2.1 Patients

Twenty‐three female and one male individuals with mild‐to‐severe facial acne vulgaris, aged 19–52 years (29.50 ± 8.83 years), were included in this retrospective study (Table 1). Individuals with comedones accompanied by few papules and pustules (mild acne), with multiple nonpainful papules and pustules (moderate acne), and with multiple papules, pustules, and rear nodules (severe acne) were included in this study. Excluded were the individuals with very severe acne characterized by abscesses and multiple nodular lesions as well as the subjects who received local or systemic treatment with corticosteroids, retinoids, or nonsteroidal anti‐inflammatory drugs less than 3 months before the beginning of the treatment course with TF‐LDM. All subjects were organized in two groups: Group 1—patients treated with TF‐LDM of 1/3/10 MHz; Group 2—patients treated with TF‐LDM of 3/10/19 MHz. No subjects received any pharmacological or other anti‐acne treatments during the treatment course with TF‐LDM and during the whole follow‐up period.

**TABLE 1:** Subjects and their treatment parameters and baseline mGEA assessments.

Patient	Age	Device	Sessions	Follow‐up, months
1	47	Triple	12	8.0
2	24	Tri	6	1.0
3	19	Triple	7	—
4	21	Triple	12	10.0
5	29	Triple	8	—
6	34	Triple	10	7.0
7	24	Tri	6	—
8	22	Tri	2	—
9	27	Triple	5	6.0
10	45	Triple	8	
11	36	Triple	6	22.0
13	19	Tri	5	3.5
14	26	Triple	6	—
15	19	Triple	6	—
16	23	Triple	4	—
17	52	Tri	4	3.0
18	25	Tri	4	9.0
20	35	Tri	6	—
21	33	Tri	6	—
22	37	Tri	7	—
23	32	Tri	6	—
24	25	Tri	7	—

### 2.2 Study Design

**FIGURE 1:**
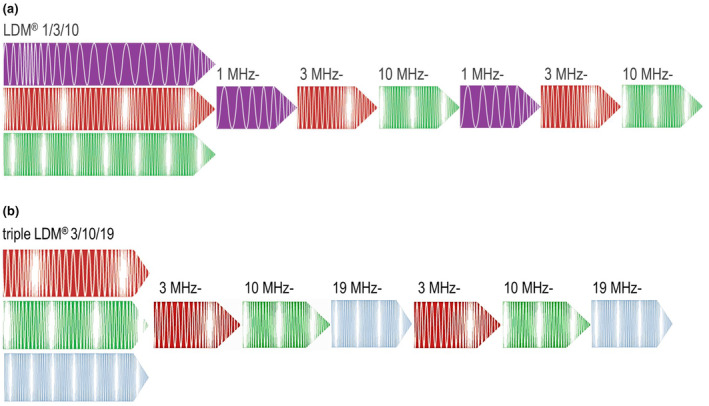
Wave formation in TF‐LDM modus: (a)—1/3/10 MHz and (b)—3/10/19 MHz.

This was a retrospective pragmatic two‐center comparative clinical study. Since the participants can clearly identify the treatment with ultrasound waves [20], neither the participants nor the study nurses or the doctors were blinded. The study was conducted with devices LDM MED Tri operating with TF‐LDM (1/3/10 MHz) and LDM Triple operating with TF‐LDM (3/10/19 MHz) (Wellcomet GmbH, Bruchsal, Germany). In LDM technology, the single ultrasound frequencies quickly (every 1–10 ms) alternate (Figure 1). Since the skin cells cannot develop the full reaction to a single mechanical impact of several milliseconds, application of LDM waves forced them to react simultaneously to all applied frequencies producing the effect that was named “biological interference.” Ultrasound waves with frequencies of 1, 3, 10, and 19 MHz have the half‐value penetration depths in the skin of approximately 30, 10, 3, and 1.5 mm, respectively; thus, application of the TF‐LDM waves of 1/3/10 and 3/10/19 MHz allows different in‐depth treatment of the inflammatory skin conditions.

Treatments were provided in two different clinics (DK Forma, St. Petersburg, Russia—with LDM Triple and TriActive, Moscow, Russia—with LDM MED Tri). Every participating center made the treatments only with one device and thus applied either TF‐LDM of 1/3/10 MHz or of 3/10/19 MHz. All patients received up to 12 treatments twice per week (Table 1). During the treatment, the whole acne lesional area was treated uniformly with a standard program “Acne papulopustulosa” of the corresponding device, which made this study to a pragmatic one. This program in device LDM MED Tri operates with the following treatment parameters: 10 MHz (0.5 W/cm^2^, 3 min)/LDM 3/10 MHz (0.5/1.0 W/cm^2^, 3 min)/LDM 1/3/10 MHz (0.5/0.7/1.0 W/cm^2^, 3 min) on each side of the face; in device LDM Triple, this program operates with parameters 19 MHz (0.5 W/cm^2^, 3 min)/LDM 10/19 MHz (0.5/0.7 W/cm^2^, 3 min)/LDM 3/10/19 MHz (0.5/0.7/1.0 W/cm^2^, 3 min) on each side of the face. Additionally, nine patients from this cohort received the follow‐up controls after the last treatment session (Table 1). Standard ultrasound gel without any additional pharmacological drugs was applied as a coupling medium during the treatment.

### 2.3 Clinical Assessment

Assessment of the acne severity was done using the facial photographs which were taken with a 15.1 megapixel digital single‐lens reflex camera Canon EOS 50D with the Macro Ring Lite MR‐14EX II flash head allowing a uniform lighting and shadow control (Canon, Japan). Frontal full face and bilateral profile views were taken using a standardized patient positioning.

The photos of all patients were evaluated at three time points—baseline (T1), on the day of the last treatment (T2), and, when available, during the follow‐up control (T3). Since this was a retrospective study and only the subjects with facial acne were involved, assessment of the acne severity was done in accordance with a modified Global Evaluation Acne (mGEA) scale [21]. The scores of 0 = *clear* (no acne lesions, eventually light erythema, or residual pigmentation); 1–2 = *almost clear* (few scattered comedones and/or papules); 3–4 = *mild* (less than half of the face is involved, few comedones and/or papules); 5–6 = *moderate* (less than half of the face is involved, multiple comedones and papules or pustules); 7–8 = *severe* (the whole face is involved, multiple comedones, papules or pustules, rare nodules); and 9–10 = *very severe* (severe inflammatory acne covering the whole face, multiple nodules) were used for grading. All assessments were provided by nine independent dermatologists, who were blinded to treatment assignments.

### 2.4 Statistical Analysis

Statistical analysis was performed using SPSS statistic software ver. 1.28 (IBM) by Noack Statistik GmbH (Bonn, Germany). Data were expressed as means ± standard deviations (SDs). The assessed acne severities were presented in absolute numbers and as a percentage of improvement compared to the corresponding time point. Since the preliminary assessment of the distribution of the mGEA scores using the Shapiro–Wilk test demonstrated a non‐normal distribution of variables, the nonparametric Wilcoxon test was applied to compare the severity grade of acne before and after TF‐LDM treatment as well as during follow‐up controls. The Mann–Whitney *U* test for independent samples was used to compare the treatment results obtained for Groups 1 and 2. The Friedman test was applied to evaluate the differences in mGEA scoring between the participating dermatologists. To avoid the biasing of results, the patients with the coefficient of variation (CV) of the baseline acne severity exceeding 33% (as assessed by all dermatologists) were excluded from further evaluation because of the high volatility of the assessed scores. A value of *p* < 0.05 was considered statistically significant.

## 3 Results

Description of the patients, their treatments, and the follow‐up information are presented in Table 1. After the preliminary assessment of the baseline mGEA and of the corresponding CV between the assessing dermatologists, patients 12 and 19 were excluded from this study since they had the CV over 33%, which indicated a high volatility of assessment results and was highly likely connected with a lower quality of the facial photographs presented for evaluation. The nonplacebo and nonsystemic effects of the treatment with very high frequency ultrasound were evidenced earlier by face splitting: Treatment effect was observed only on the treated side [19, 22].

The mean number of treatments for the total cohort of treated individuals was 6.5 ± 2.4. The mean age of 22 patients included in this study was 29.7 ± 9.1; there was no statistically significant difference between the number of treatments or mean ages between the patients from Groups 1 and 2.

The mean values of mGEA(T1), mGEA(T2) as well as the relative improvement of mGEA between these time points for every patient are presented in Table 2.

**TABLE 2:** Treatment outcomes on the day of the last treatment.

Patient	Device	mGEA(T1)	mGEA(T2)	mGEA improvement (%)
1	Triple	4.56 ± 0.48	1.00 ± 0.00	78.05
2	Tri	4.89 ± 0.33	0.56 ± 0.50	88.64
3	Triple	8.78 ± 0.43	4.56 ± 0.50	48.10
4	Triple	8.22 ± 0.43	2.33 ± 0.47	71.62
5	Triple	3.78 ± 0.43	0.67 ± 0.47	82.35
6	Triple	4.22 ± 0.66	0.78 ± 0.42	81.58
7	Tri	5.44 ± 1.00	1.44 ± 0.68	73.47
8	Tri	5.00 ± 0.00	2.00 ± 0.00	60.00
9	Triple	4.11 ± 0.60	0.67 ± 0.47	83.78
10	Triple	4.33 ± 0.43	1.11 ± 0.31	74.36
11	Triple	6.33 + 0.87	2.11 ± 0.57	66.67
13	Tri	5.67 ± 0.70	0.00 ± 0.00	100.00
14	Triple	3.67 ± 0.43	0.56 ± 0.50	84.85
15	Triple	4.22 ± 0.43	1.78 ± 0.42	57.89
16	Triple	4.22 ± 0.83	1.44 ± 0.50	65.79
17	Tri	5.22 ± 0.33	1.56 ± 0.68	70.21
18	Tri	6.67 ± 1.12	2.00 ± 0.47	70.00
20	Tri	3.67 ± 1.11	0.78 ± 0.42	78.79
21	Tri	2.78 ± 0.43	1.00 ± 0.00	64.00
22	Tri	5.44 ± 1.32	2.89 ± 1.20	46.94
23	Tri	2.00 ± 0.00	0.00 ± 0.00	100.00
24	Tri	6.89 ± 1.17	1.78 ± 0.42	74.19

**FIGURE 2:**
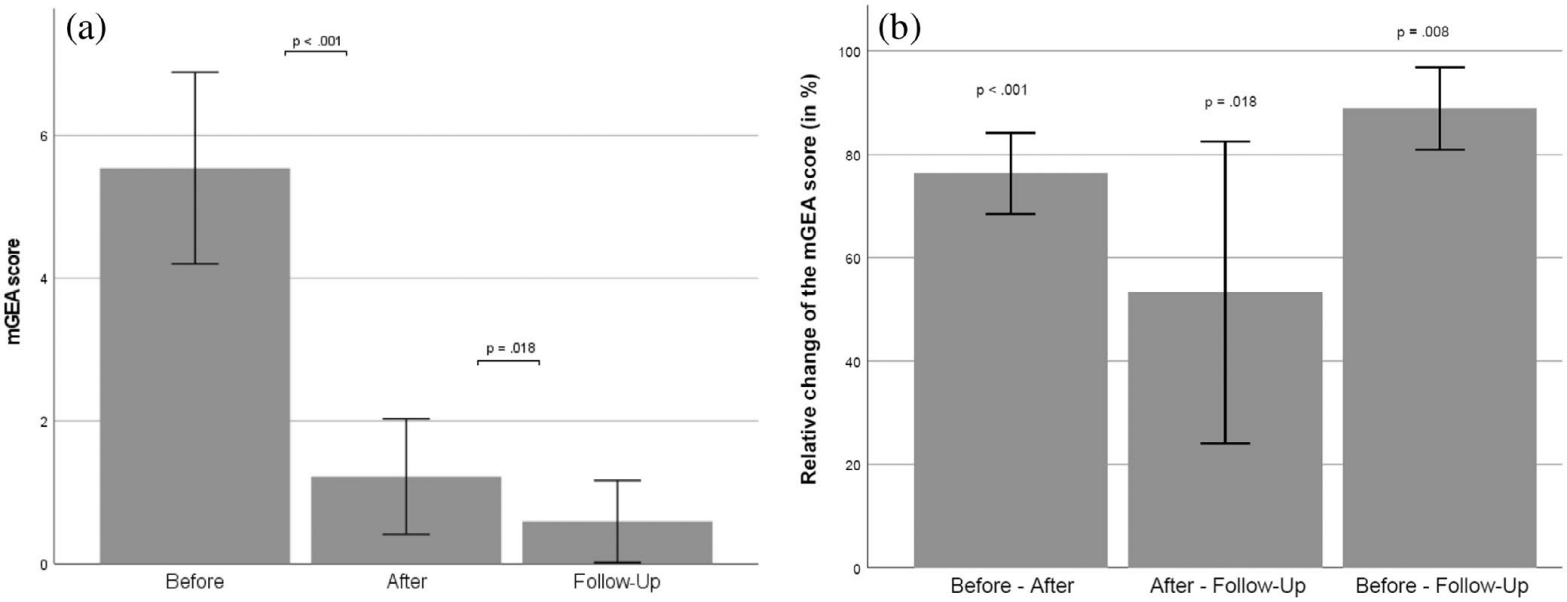
Mean mGEA scores (a) and relative improvement of mGEA (b) evaluated at the time points T1 (baseline), T2 (after the treatment course), and T3 (follow‐up). Statistical differences between mGEA(T1) and mGEA(T2) as well as between mGEA(T2) and mGEA(T3) are highly significant.

The mean mGEA(T1) and mGEA(T2) scores assessed for all patients were 5.01 ± 1.63 and 1.41 ± 1.03, respectively. Changes between mGEA(T1) and mGEA(T2) were highly statistically significant for each of 22 patients as well as for the whole cohort of patients (*p* < 0.01) (Figure 2). The average relative improvement of the mGEA scoring between the time points T1 and T2 across all patients was 73.69% ± 13.90%. There was no statistically significant difference in improvement of the mGEA scores between the groups.

The assessments of mGEA(T3) during the follow‐up controls and their differences to the baseline mGEA(T1) are presented in Table 3.

**TABLE 3:** Treatment outcomes during the follow‐up controls.

Patient	Device	mGEA(T1)	mGEA(T3)	mGEA improvement (%)
1	Triple	4.56 ± 0.48	1.00 ± 0.00	78.05
2	Tri	4.89 ± 0.33	0.22 ± 0.42	95.45
4	Triple	8.22 ± 0.43	1.56 ± 0.50	81.08
6	Triple	4.22 ± 0.66	0.22 ± 0.42	94.74
9	Triple	4.11 ± 0.60	0.22 ± 0.42	94.59
11	Triple	6.33 + 0.87	0.67 ± 0.47	89.47
13	Tri	5.67 ± 0.70	0.00 ± 0.00	100.00
17	Tri	5.22 ± 0.33	0.00 ± 0.00	97.87
18	Tri	6.67 ± 1.12	0.11 ± 0.31	80.00

The mean mGEA(T3) score for nine patients with follow‐up was 0.59 ± 0.57. These changes were highly statistically significant (*p* < 0.01) for each of these patients compared to their baseline mGEA(T1), which confirms a long‐lasting effect of the TF‐LDM treatment on the acne skin. The average improvements of the mGEA(T3) scores across all patients was 90.14% ± 8.35% compared to their mGEA(T1) values; improvement of mGEA scores between the time points T2 and T3 was 53.26% ± 29.24% and demonstrated a further significant (*p* < 0.02) improvement of the acne skin after the treatment course with TF‐LDM was completed (Figure 2).

**FIGURE 3:**
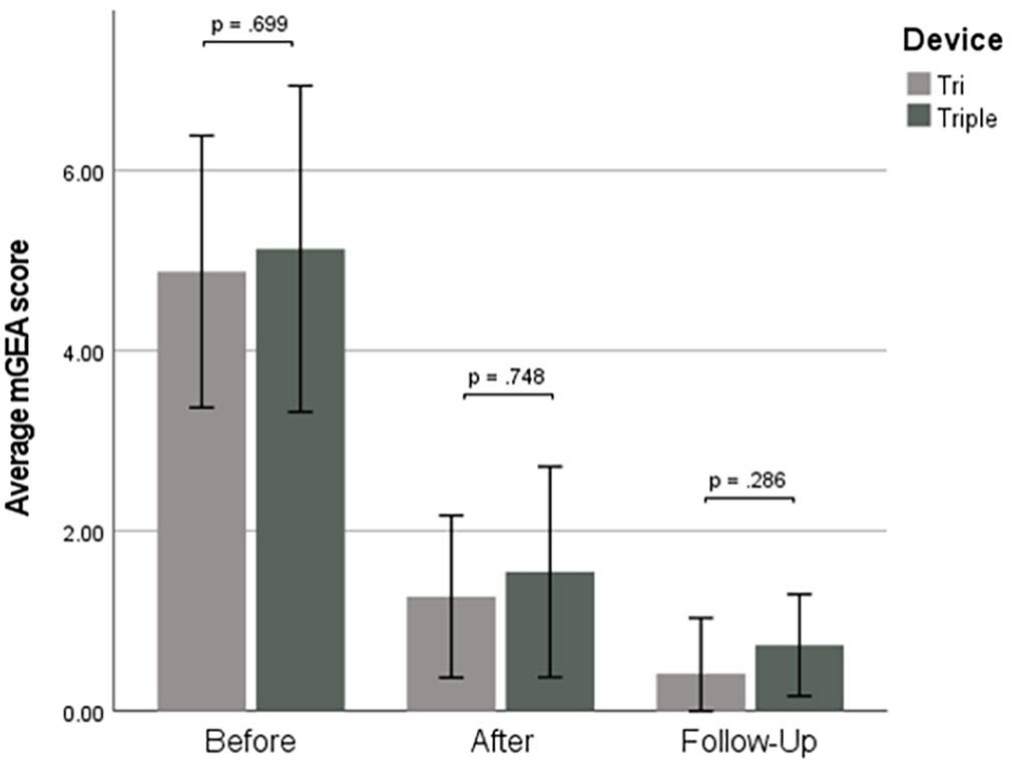
Mean mGEA values at the time points T1, T2, and T3 for the patients from Groups 1 and 2. There were no statistically significant differences between the treatment groups at any time point.

**FIGURE 4:**
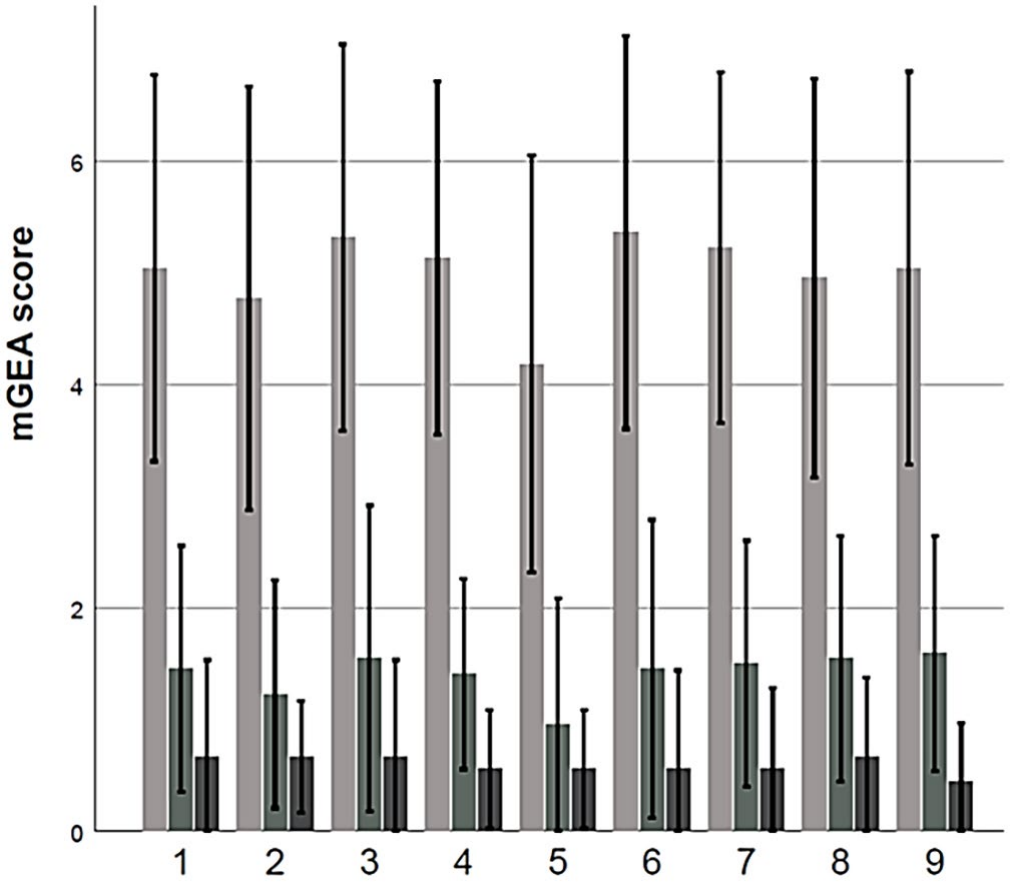
Mean mGEA values evaluated by single dermatologists at the time points T1, T2, and T3.

There was no statistically significant difference in skin improvement between Groups 1 and 2, which confirms that both TF‐LDM of 1/3/10 MHz and 3/10/19 MHz can effectively improve the acne skin (Figure 3). Also, application of the Friedman test revealed no statistically significant differences in the skin improvement as assessed by different doctors, which confirms the robustness of the applied scoring procedure (Figure 4).

**FIGURE 5:**
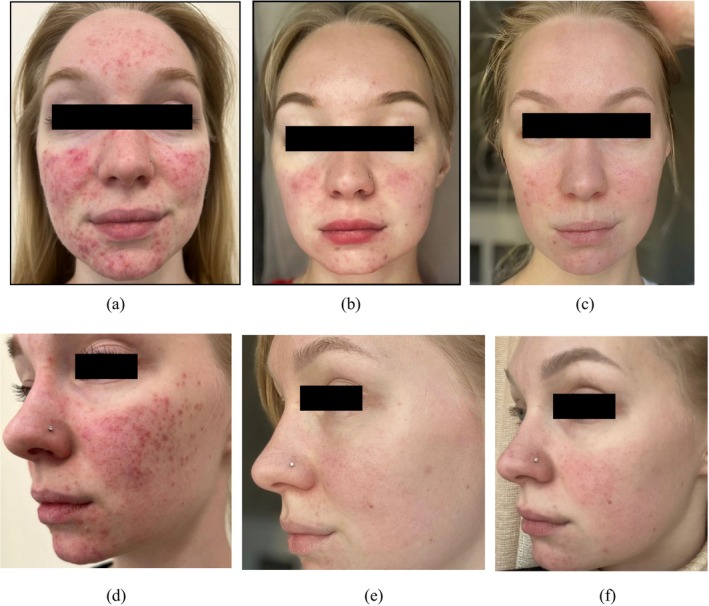
Severe acne vulgaris (a, d) before, (b) after eight treatment sessions with TF‐LDM (3/10/19 MHz), (c,e) follow‐up 10 months after 12 treatment sessions, and (f) follow‐up after 19.5 months.

**FIGURE 6:**
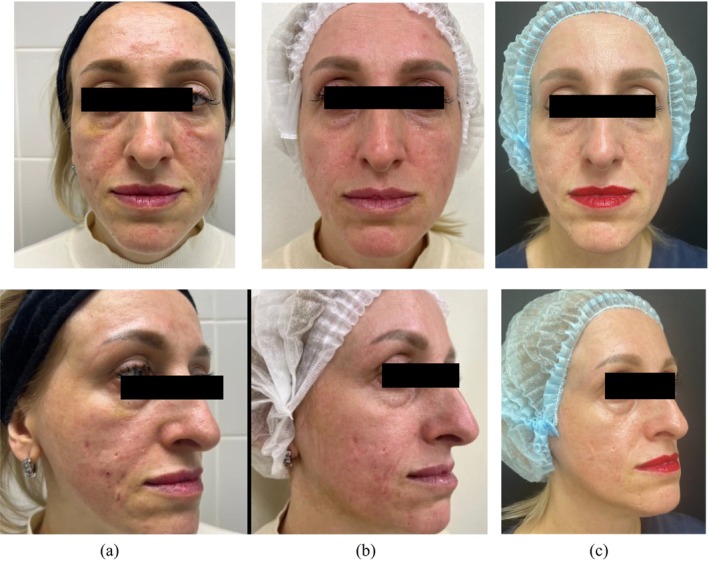
Moderate acne vulgaris with acne scars: (a) before, (b) after 12 treatments with TF‐LDM (3/10/19 MHz, and (c) follow‐up after 8.0 months.

**FIGURE 7:**
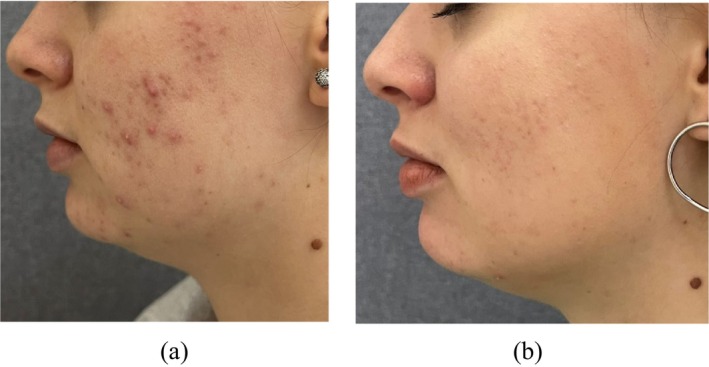
Moderate acne vulgaris: (a) before and (b) after six treatments with TF‐LDM (1/3/10 MHz).

Some treatment results after the application of the TF‐LDM waves are presented in Figures 5, 6, 7.

No side effects were observed during and after treatment with TF‐LDM of both 1/3/10 and 3/10/19 MHz; these treatments were pain‐free, well‐tolerated, and highly accepted by patients. Some patients claimed a short‐term erythema directly after the treatment that disappeared within 1 h.

## 4 Discussion

As it was demonstrated in previous studies, dual‐frequency LDM waves with frequencies of 3/10 MHz can be used for the treatment of different inflammatory and hyperproliferative skin conditions [18, 19, 22, 23]. Results obtained in the present study demonstrate a significant and long‐lasting skin improvement in patients with mild‐to‐severe acne vulgaris after treatment with TF‐LDM waves with frequencies of 1/3/10 or 3/10/19 MHz. The average improvement of the acne skin observed in this study after 6.5 ± 2.4 treatments with TF‐LDM waves was more than 73% on the day of the last treatment session and was further increased during the follow‐up period to about 90%. This grade of improvement assessed by nine independent dermatologists in accordance with a mGEA scale was highly statistically significant compared to the baseline state of acne skin in the same patients. This means that the repair and regenerative processes induced by the application of the TF‐LDM waves continue after completing the treatment course, making the treatment results remarkably stable. Patients treated with TF‐LDM waves of 1/3/10 or 3/10/19 MHz demonstrated no statistically significant differences in treatment results, which confirms that both TF‐LDM methods are effective in the improvement of acne skin.

Remarkably, even patients with high baseline mGEA scores demonstrated stable treatment results or further skin improvement during the follow‐up period. For example, patient #1 with a baseline mGEA score of 4.56 ± 0.48 demonstrated reduction of this score to 1.00 ± 0.00 after 12 treatments with TF‐LDM (3/10/19 MHz), which remained constant at least after 8 months' follow‐up. Patient #4 with a baseline mGEA score of 8.22 ± 0.43 had a reduction of this score to 2.33 ± 0.47 after 12 treatments with TF‐LDM (3/10/19 MHz) and its further improvement to 1.56 ± 0.50 after 10 months follow‐up. Patient #11 with a baseline mGEA score of 6.33 ± 0.87 demonstrated a reduced mGEA score of 2.11 + 0.57 after six treatments with TF‐LDM (3/10/19 MHz) and the mGEA score of 0.67 ± 0.47 after 22.0 months follow‐up.

This study has several limitations. The first one concerns the follow‐up controls in the groups treated with TF‐LDM (1/3/10 MHz) and TF‐LDM (3/10/19 MHz): Since only 9 of 22 patients included in this study had follow‐up controls, it was not possible to compare the follow‐up outcomes in these treatment groups. The second limitation is connected with the determination of the remission time, which obviously demands longer follow‐up controls since the follow‐up times used in this study did not reveal a significant worsening of treatment outcomes in single patients. The third limitation is connected with the pragmatic character of this study connected with the application of the standard programs available in applied devices; this did not allow to investigate the role of treatment parameters in the effect of TF‐LDM on acne skin. The fourth limitation was connected with a relatively small sample size and retrospective type of the study which did not allow to provide a reliable statistical evaluation to answer the question how the number of treatment sessions influence the treatment outcomes. Additional study will be needed to address these open questions.

## 5 Conclusions

Triple‐frequency LDM technology of 1/3/10 or 3/10/19 MHz allows a highly statistically significant skin improvement in patients with a mild‐to‐severe acne. Treatment outcomes are long‐lasting; moreover, acne skin demonstrates a further spontaneous improvement after completing the treatment course. The method is pain‐free, well‐tolerated, and highly accepted by patients and demonstrates no significant side effects.

## Ethics Statement

Authors declare that human ethics approval was not needed for this study. The study was conducted in compliance with the principles set forth in the Declaration of Helsinki.

## Conflicts of Interest

I.L.K. is the CEO of Wellcomet GmbH. He was involved neither in clinical treatments nor in the assessment of treatment results. Wellcomet GmbH provided support in the form of salaries for I.L.K., but did not have any additional role in the decision to publish or the preparation of this manuscript. The commercial affiliation of I.L.K. with Wellcomet GmbH does not alter the adherence to all journal policies on sharing data and materials. Other authors declare no conflict of interest.

## Data Availability

The data that support the findings of this study are available on request from the corresponding author. The data are not publicly available due to privacy or ethical restrictions.
